# Lack of efficacy of a partial adenosine A1 receptor agonist in neuropathic pain models in mice

**DOI:** 10.1007/s11302-021-09806-6

**Published:** 2021-07-27

**Authors:** Katharina Metzner, Tilman Gross, Annika Balzulat, Gesine Wack, Ruirui Lu, Achim Schmidtko

**Affiliations:** Institute of Pharmacology and Clinical Pharmacy, Goethe University Frankfurt, 60438 Frankfurt am Main, Germany

**Keywords:** Neuropathic pain, Adenosine A_1_ receptor, Partial agonist, Pain behavior, In situ hybridization, Patch-clamp

## Abstract

Previous studies suggest that adenosine A_1_ receptors (A_1_R) modulate the processing of pain. The aim of this study was to characterize the distribution of A_1_R in nociceptive tissues and to evaluate whether targeting A_1_R with the partial agonist capadenoson may reduce neuropathic pain in mice. The cellular distribution of A_1_R in dorsal root ganglia (DRG) and the spinal cord was analyzed using fluorescent in situ hybridization. In behavioral experiments, neuropathic pain was induced by spared nerve injury or intraperitoneal injection of paclitaxel, and tactile hypersensitivities were determined using a dynamic plantar aesthesiometer. Whole-cell patch-clamp recordings were performed to assess electrophysiological properties of dissociated DRG neurons. We found A_1_R to be expressed in populations of DRG neurons and dorsal horn neurons involved in the processing of pain. However, administration of capadenoson at established in vivo doses (0.03–1.0 mg/kg) did not alter mechanical hypersensitivity in the spared nerve injury and paclitaxel models of neuropathic pain, whereas the standard analgesic pregabalin significantly inhibited the pain behavior. Moreover, capadenoson failed to affect potassium currents in DRG neurons, in contrast to a full A_1_R agonist. Despite expression of A_1_R in nociceptive neurons, our data do not support the hypothesis that pharmacological intervention with partial A_1_R agonists might be a valuable approach for the treatment of neuropathic pain.

## Introduction

Traumatic injuries, surgical insults and damages of peripheral nerves often lead to neuropathic pain, a chronic debilitating disease that affects 7–10% of the general population and is associated with great impairment of quality of life [1]. However, more than half of neuropathic pain patients report inadequate pain relief with currently available medications, and these are often associated with severe dose-limiting side effects. Therefore, there is a large unmet therapeutic need for effective and safe treatment of neuropathic pain [2–4].

Neuropathic pain is associated with multiple alterations in the peripheral and central nervous system [5, 6]. Accumulating evidence indicates that the nucleoside adenosine contributes to the processing of neuropathic pain [7, 8]. In general, adenosine interacts with four G protein-coupled receptors, A_1_R, A_2A_R, A_2B_R and A_3_R, which in turn affect the activity of various ion channels and enzymes [9]. Among the adenosine receptors, A_1_R has gained interest in pain research. Previous studies reported that A_1_R is expressed in both peripheral and central sites of the nociceptive system, although the cellular distribution remains controversial [10–13]. Several lines of evidence indicate the functional contribution of A_1_R to neuropathic pain processing. For example, mice lacking A_1_R globally demonstrated increased pain behaviors in models of neuropathic pain [14]. Increasing adenosine levels by delivery of ectonucleotidases that dephosphorylate adenosine 5′-monophosphate to adenosine is associated with potent, long-lasting, and A_1_R-dependent antinociceptive effects [15, 16]. Furthermore, administration of A_1_R agonists such as N^6^-cyclopentyladenosine (CPA) or 5’-chloro-5’deoxy-( ±)-ENBA (Cl-ENBA) ameliorated neuropathic pain in various animal models (for review, see [7]).

Although numerous full A_1_R agonists have been developed, clinical applications of these agents have been hampered by unintended pharmacological effects including sedation, motor impairment, bradycardia and atrioventricular blocks [17, 18]. These unwanted effects can be overcome by partial A_1_R agonists, which trigger only some of the physiological responses of receptor activation depending on endogenous adenosine levels and on receptor reserve in different tissues [19]. Partial A_1_R agonists might therefore hypothetically ameliorate neuropathic pain in an effective and safe manner. Among the selective and potent partial A_1_R agonists is capadenoson, which belongs to the non-adenosine dicyanopyridine class of compounds. Capadenoson shows EC_50_ values of 0.1 nM on A_1_R, a selectivity factor of 1800 and 900 versus A_2A_R and A_2B_R, respectively, and no significant activity on A_3_R [19]. Furthermore, it exhibits good pharmacokinetic parameters with sufficient bioavailability after oral administration [18, 19]. The primary objectives of the study were to characterize the cellular distribution of A_1_R in nociceptive tissues and to investigate whether targeting A_1_R using the partial agonist capadenoson might inhibit neuropathic pain in mice.

## Material and methods

### Animals

All experiments were performed in C57BL/6 N mice of either sex (6–12 weeks old) obtained from Charles River Laboratories (Sulzfeld, Germany). Animals were housed on a 12 h light/dark cycle with access to food and water ad libitum. All behavioral studies were carried out by observers blinded for treatment of the animals. All experiments were ethically reviewed and approved by our local Ethics Committee for Animal Research (Regierungspräsidium Darmstadt, Germany). They adhered to the IASP (International Association for the Study of Pain) and ARRIVE (Animal Research: Reporting on In Vivo Experiments) guidelines and conformed to Directive 2010/63/EU. All efforts were made to minimize animal suffering and to reduce the number of animals used.

### Neuropathic pain models

The spared nerve injury (SNI) model [20] was used to investigate neuropathic pain behavior after surgically induced peripheral nerve injury. Animals were treated with carprofen (5 mg/kg, s.c.) 30 min prior to surgery to provide perioperative and postoperative analgesia. Under isoflurane anesthesia, two branches of the sciatic nerve were ligated and cut distally, leaving the sural nerve intact. This procedure leads to a hypersensitivity of the lateral surface (sural nerve skin area) of the affected hindpaw.

The paclitaxel model of neuropathy was used to mimic chemotherapy-induced neuropathic pain behavior. Animals received four i.p. injections of 1 mg/kg paclitaxel on days 0, 2, 4 and 6 (cumulative dose 4 mg/kg; [21]. Paclitaxel (Sigma Aldrich, Germany) was dissolved in a vehicle composed of Cremophor EL and absolute ethanol (1:1) and was further diluted in 0.9% NaCl [22, 23].

Mechanical sensitivity of the hindpaw was measured using a Dynamic Plantar Aesthesiometer (Ugo Basile, Italy). This device pushes a thin steel rod against the plantar surface of the paw from beneath, and automatically stops and records the latency time until the animal withdraws the paw. The force increased constantly from 0 to 5 g in 10 s (ramp 0.5 g/s) and remained at 5 g for an additional 10 s [23–25]. The paw withdrawal latency was calculated as the mean of 4–5 consecutive measurements with at least 20 s in between. Baseline measurements of mechanical sensitivity were performed 2 and 1 days before SNI surgery or paclitaxel injections. To ensure full development of neuropathic pain, mechanical sensitivity of the hindpaw was determined 13 or 20 days after SNI and 6 days after the last paclitaxel injection. One day thereafter, capadenoson (provided by Bayer AG, Germany, and purchased from MedChemExpress, USA), pregabalin (Bertin, France and Neuraxpharm, Germany), or vehicle (85% PEG400 and 15% glycerol; both from Carl Roth, Germany) were administered by oral gavage, whereas in another set of experiments capadenoson, N-Bicyclo[2.2.1]hept-2-yl-5'-chloro-5'-deoxyadenosine (CL-ENBA; Tocris, UK) or vehicle (60% PEG400 in water; Carl Roth, Germany) were administered by tail vein injection. The mechanical sensitivity of the ipsilateral hindpaw was determined over 24 h after drug administration.

### In situ hybridization

Mice were killed by CO_2_ inhalation and perfused with 4% formaldehyde (PFA) in phosphate-buffered saline (PBS) for 5 min. Lumbar (L4-L5) spinal cords and lumbar (L4-L5) DRGs were dissected, post-fixed in PFA for 10 min, incubated in 20% sucrose in PBS overnight, and embedded in tissue freezing medium (Leica, Germany). Cryostat sections were cut at a thickness of 14 µm on a CryoStar NX50 device (Thermo Fisher Scientific, Germany). In situ hybridization (ISH) was performed using a QuantiGene ViewRNA Tissue Assay (Thermo Fisher Scientific, Germany) according to the manufacturer’s instructions and as previously described [26]. Briefly, probes for mouse Adora1 (diluted 1:40; NM_001039510.2, type 1 probe set, catalog # VB1-19,627, Thermo Fisher, Germany), Rbfox3 (diluted 1:40; NM_001039167.1, type 6 probe set, catalog # VB6-18,012) and scramble control (1:40; catalog # VF1-17,155) were incubated overnight at 40 °C (Thermobrite; Leica, Germany) followed by consecutive incubation with PreAmplifier Mix QT, Amplifier Mix QT, an alkaline phosphatase labeled probe against the Amplifier, AP Enhancer Solution, and Fast Red Substrate. Finally, sections were mounted with Fluoromount G (Southern Biotech, USA) or further processed for subsequent immunostaining.

In immunostaining experiments after in situ hybridization, sections were blocked in 10% normal goat serum (NGS), 3% bovine serum albumin (BSA) and 1% Triton X-100 in PBS for 1 h and incubated with primary antibodies overnight using rabbit anti-NF200 (1:2000; # N4142, Sigma-Aldrich, Germany) and rabbit anti-CGRP (1:800, # PC205C, Calbiochem, Germany). After rinsing in PBS, sections were incubated with secondary antibodies conjugated with Alexa Fluor 488 (Invitrogen/Life Technologies, USA) for 2 h at room temperature. For staining with *Griffonia simplicifolia* isolectin B4 (IB4), sections were incubated with AF488-labelled IB4 (# I21411, Invitrogen/Life Technologies, USA; dissolved 1:300 in PBS buffer containing 1 mM CaCl_2_, 1 mM MgCl_2_, 1 mM MnCl_2_, and 0.2% Triton X-100, pH 7.4) for 1 h at room temperature. Slides were coverslipped with Fluoromount G (Southern Biotech, USA). Images were taken using an Eclipse Ni-U microscope equipped with a monochrome DS-Qi2 camera (both from Nikon, Germany) and pseudocolored with the NIS Elements software (Nikon, Germany).

### Cell counting

For quantification of A_1_R mRNA-positive sensory and dorsal horn neuron populations, serial sections of lumbar DRGs (L4-L5) and the lumbar spinal cord (L4–L5) from 3 mice were cut (14 µm). Per animal, ≥ 3 DRG sections at least 100 µm apart were counted manually (4837 cells in total). Only cells showing clear staining signals above background level, with a threshold set based on scramble control hybridization, were included. The percentage of CGRP-, IB4- and NF200-positive neurons is expressed as a proportion of marker-positive cells per total number of A_1_R-positive neurons. For calculation of the percentage of A_1_R-positive DRG neurons, the total number of DRG neuron somata was counted based on their autofluorescence visualized in the FITC channel.

### DRG neuron culture

Mice (4–8 weeks old) were killed by CO_2_ inhalation and lumbar DRGs (L4-L5) were excised and transferred to HBSS (Thermo Fisher Scientific, USA). Following treatment with 2.5 U/ml dispase II and 500 U/ml collagenase IV (both from Roche, Switzerland) for 90 min and 0.05% Trypsin/EDTA (Thermo Fisher Scientific, USA) for 10 min, isolated cells were transferred onto coverslips coated with poly-d-lysine (250 μg/ml, Millipore, USA) and cultured in neurobasal medium supplemented with B27 (Thermo Fisher Scientific, USA), 100 μg/ml streptomycin and penicillin (Roth, Germany) at 37 °C and 5% CO_2_. Cells were used for experiments within 24 h after plating.

### Electrophysiological recordings

Whole-cell voltage-clamp recordings on DRG neurons were performed at room temperature (20–22 °C), using an HEKA EPC 9 amplifier and Patchmaster software (HEKA Electronics, Germany). Offline analysis was performed using the Fitmaster software (HEKA Electronics, Germany) and GraphPad Prism 8. Micropipettes (3–5 MΩ) were pulled from borosilicate glass (Science Products, Germany) with a conventional micropipette puller (Model P-97, Sutter Instruments, USA). Potassium currents were measured by continuous perfusion of the external solution with clamp steps of 500 ms between -100 and + 120 mV starting from a 1000 ms prepulse at − 100 mV. The holding potential was − 70 mV. Current densities were normalized to the cell capacitance (pA/pF). The pipette solution contained (mM): KCl 140, MgCl_2_ 2, EGTA 5, HEPES 10, MgATP 2, TrisGTP 1, pH 7.4 adjusted with KOH. The external solution contained (mM): NaCl 140, KCl 5, CaCl_2_ 2, MgCl_2_ 2, HEPES 10, pH 7.4 adjusted with NaOH. Capadenoson or CPA (Sigma-Aldrich, Germany), solved in external solution with a final concentration of 100 nM, were added to the bath without a continuous perfusion. Potassium currents were measured within 10 min after drug addition.

### Statistical analysis

GraphPad Prism 8 software was used for statistical analysis. A two-way repeated-measures ANOVA with Bonferroni post hoc test was used to assess statistical significance. Changes with *p* < 0.05 were considered to be significant. All data are presented as mean ± SEM.

## Results

### Cellular distribution of adenosine A_1_ receptors in dorsal root ganglia and the spinal cord

**Fig. 1:**
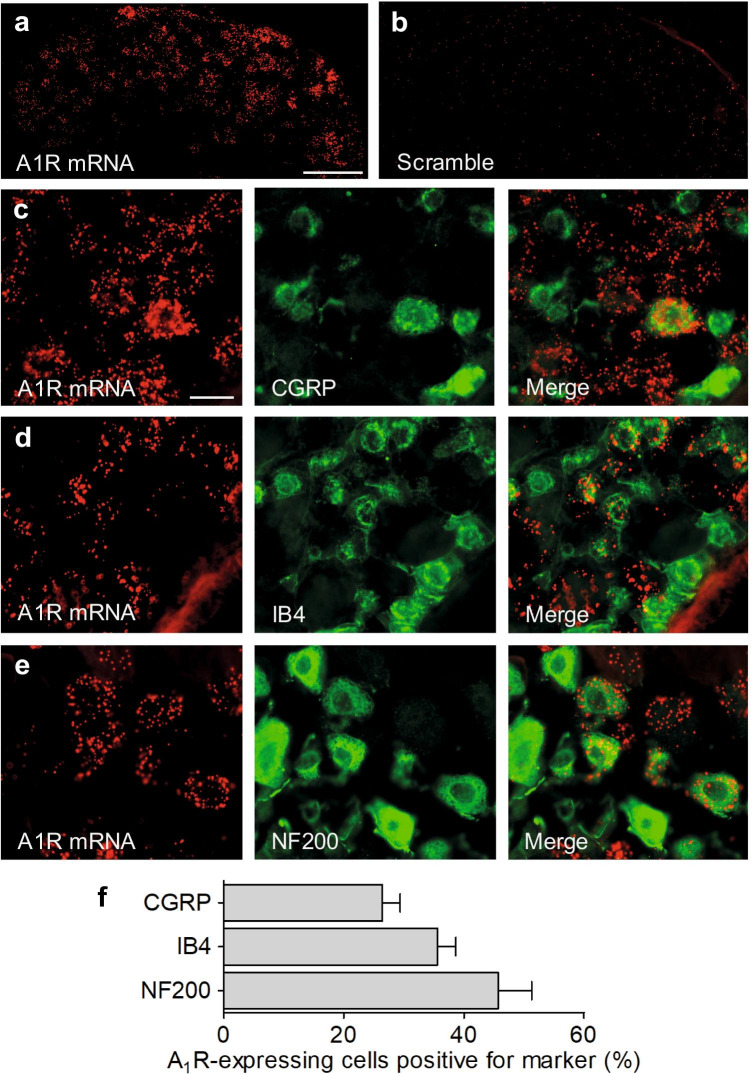
Distribution of adenosine A_1_ receptors (A_1_R) in dorsal root ganglia (DRG). **a** Fluorescent in situ hybridization detected A_1_R mRNA in mouse DRGs. **b** No hybridization signal was detected using a scramble control probe. **c**–**e** Fluorescent in situ hybridization of A_1_R mRNA combined with immunostaining of calcitonin gene-related peptide (CGRP; c), binding of isolectin B4 (IB4; d), or immunostaining of neurofilament-200 (NF200; e) revealed that A_1_R mRNA is expressed in populations of peptidergic and non-peptidergic C fibers and myelinated DRG neurons, respectively. **f** Quantitative summary of DRG neuron populations expressing A_1_R (2061 cells counted; n = 3 animals). Scale bars: 100 µm (a), 25 µm (c)

We first investigated the cellular distribution of A_1_R mRNA in DRGs and the spinal cord using fluorescent in situ hybridization. In DRGs, we detected abundant hybridization signals (Fig. 1a), which were seen primarily in neuronal somata. Cell counting revealed that 60.7 ± 2.4% of DRG neurons express A_1_R mRNA. No hybridization signals were detected using a scramble control probe (Fig. 1b). To analyze the localization of A_1_R in DRG neuron subpopulations we combined in situ hybridization of A_1_R mRNA with immunostaining for established markers (Fig. 1c-f). Out of the A_1_R-positive neurons, 26.4 ± 2.9% coexpressed CGRP, a marker of peptidergic C fiber neurons, and 35.6 ± 3.0% bound IB4, a marker of non-peptidergic C fiber neurons. Furthermore, 45.7 ± 5.6% of A_1_R-positive neurons co-expressed NF200, which stains myelinated DRG neurons. These data suggest that A_1_R are expressed in both unmyelinated and myelinated DRG neurons of naive mice.

**Fig. 2:**
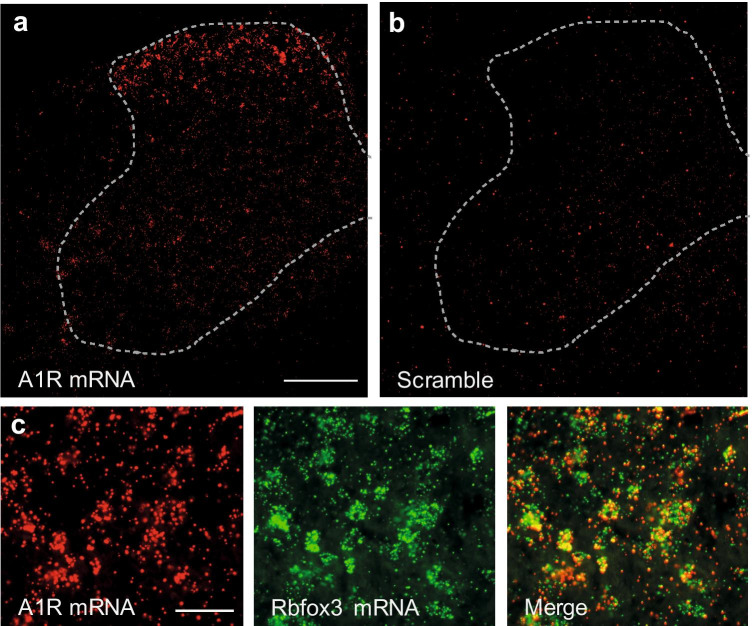
Distribution of adenosine A_1_ receptors (A_1_R) in the spinal cord. **a** Fluorescent in situ hybridization detected A_1_R mRNA primarily in the dorsal horn of mouse spinal cord. **b** No hybridization signal was detected using a scramble control probe. **c** Double-labeling in situ hybridization of A_1_R mRNA with mRNA of the neuronal marker Rbfox3 in the dorsal horn shows that A_1_R is mainly expressed by neurons. Scale bars: 500 µm (a), 50 µm (c)

In the spinal cord, A_1_R mRNA was enriched in cells of the dorsal horn (Fig. 2a). No specific hybridization signal was detected using scramble control, as expected (Fig. 2b). Double-labeling in situ hybridization of A_1_R with the neuronal marker Rbfox3 (which produces the ‘neuronal nuclei’ antigen NeuN) revealed that virtually all A_1_R-positive cells co-express Rbfox3 (Fig. 2c), suggesting that A_1_R is mainly expressed in neurons in the dorsal horn of the spinal cord. Together, the expression of A_1_R in DRG neurons and dorsal horn neurons further supports its contribution to pain processing.

### Treatment with capadenoson does not affect neuropathic pain behavior in mice

**Fig. 3:**
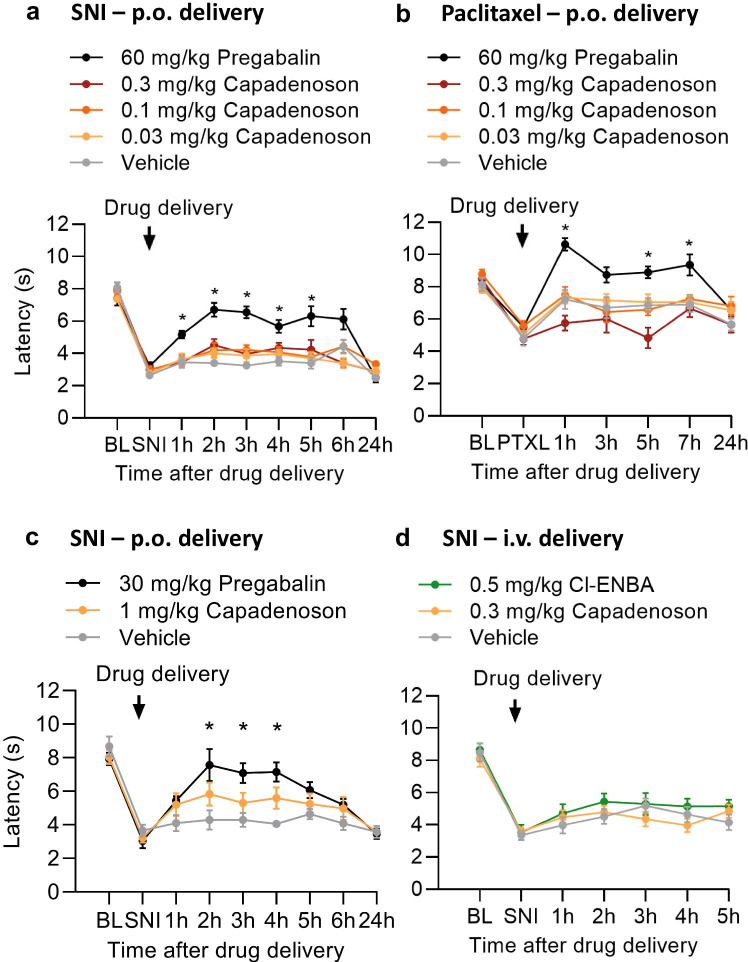
Neuropathic pain behavior in mice is not affected by capadenoson treatment. **a** In the spared nerve injury (SNI) model, neuropathic pain was induced by surgery. Fourteen days thereafter, a mechanical hypersensitivity of the affected hindpaw (determined using a Dynamic Plantar Aesthesiometer) was detected in all mice. Then animals were orally treated with vehicle (85% PEG400 / 15% glycerol, n = 13), 0.03 mg/kg capadenoson (n = 13), 0.1 mg/kg capadenoson (n = 9), 0.3 mg/kg capadenoson (n = 12), or 60 mg/kg pregabalin (n = 12) and the mechanical sensitivity was assessed over 24 h. Note that pregabalin inhibited the neuropathic pain behavior, whereas capadenoson was not effective. **b** In the paclitaxel model, neuropathic pain was induced by four i.p. injections of paclitaxel on day 0, 2, 4 and 6. Seven days after the last paclitaxel injection, 43% of treated animals developed a significant mechanical hypersensitivity. These animals were orally treated with vehicle (85% PEG400/15% glycerol, n = 11), 0.03 mg/kg capadenoson (n = 13), 0.1 mg/kg capadenoson (n = 13), 0.3 mg/kg capadenoson (n = 12), or 60 mg/kg pregabalin (n = 12) and the mechanical sensitivity was assessed over 24 h. Similar to the spared nerve injury model, pregabalin inhibited the paclitaxel-induced neuropathic pain behavior, whereas capadenoson was not effective. **c**–**d** In a separate cohort of mice, neuropathic pain was induced by SNI surgery. **c** At day 14 after SNI, the animals were orally treated with vehicle (85% PEG400/15% glycerol; n = 11), 1 mg/kg capadenoson (n = 11), or 30 mg/kg pregabalin (n = 10), and the mechanical sensitivity was assessed over 24 h. Pregabalin significantly inhibited the neuropathic pain behavior, whereas the effects of capadenoson were not significant. **d** At day 21 after SNI, the animals were i.v. treated by tail vein injection with vehicle (60% PEAG400 in water; n = 11), 0.3 mg/kg capadenoson (n = 11), or 0.5 mg/kg Cl-ENBA (n = 10) and the mechanical sensitivity was assessed over 5 h. Neither capadenoson nor Cl-ENBA did affect SNI-induced mechanical hypersensitivity. Data are presented as mean ± SEM. *p < 0.05, comparing drug treated and vehicle treated mice. Abbreviations used on the x-axis: BL: baseline sensitivity in naive animals; SNI: spared nerve injury-induced hypersensitivity before drug delivery; PTXL: paclitaxel-induced hypersensitivity before drug delivery

We next explored whether pharmacological activation of A_1_R might ameliorate neuropathic pain. For that purpose we tested whether treatment with the partial A_1_R agonist capadenoson affects mechanical hypersensitivity in two models of neuropathic pain in mice, i.e. the spared nerve injury (SNI) model of peripheral nerve injury and the paclitaxel model of chemotherapy-induced neuropathic pain. In a first set of experiments, we administered capadenoson perorally (p.o.) at three doses (0.03, 0.1 and 0.3 mg/kg). We chose these doses because capadenoson at 0.03, 0.1 and 0.3 mg/kg p.o. previously showed dose-dependent efficacy in a cardioprotection model in mice (personal communication from Cardiovascular Research, Bayer AG; data are not to be disclosed), and capadenoson at 0.3 mg/kg p.o. reduced overnight running distance on a running wheel in mice [18]. As shown in Fig. 3a, the SNI surgery induced a mechanical hypersensitivity of the affected hindpaw 13 days after SNI in all animals, as expected. One day thereafter, mice were p.o. treated with capadenoson, pregabalin or vehicle. However, treatment with capadenoson at all three doses did not significantly alter mechanical hypersensitivity over 24 h compared to the vehicle-treated group (Fig. 3a). By contrast, the positive control pregabalin (60 mg/kg [23, 27]), significantly ameliorated the mechanical hypersensitivity, confirming that the SNI-induced neuropathic pain behavior is responsive to standard analgesic treatment.

We then investigated whether capadenoson treatment may inhibit chemotherapy-induced neuropathic pain in the paclitaxel model. Four i.p. injections of 1 mg/kg paclitaxel (on days 0, 2, 4 and 6) resulted in a significant mechanical hypersensitivity (determined 6 days after the last paclitaxel injection) in 43% of all paclitaxel-injected animals. One day thereafter, mice showing a significant mechanical hypersensitivity were p.o. treated with capadenoson (0.03, 0.1 and 0.3 mg/kg), pregabalin (60 mg/kg), or vehicle. Similar to the SNI model, the delivery of capadenoson at three doses did not result in significant changes of paclitaxel-induced mechanical hypersensitivity as compared to vehicle (Fig. 3b). We observed a tendency of increased hypersensitivity after administration of 0.3 mg/kg capadenoson, which however was not significant. By contrast, treatment with pregabalin significantly inhibited the hypersensitivity (Fig. 3b). Together, these data suggest that treatment with the partial A_1_R agonist capadenoson at doses up to 0.3 mg/kg p.o. does not significantly affect neuropathic pain behavior in mice.

In a separate cohort of mice, we tested capadenoson at a higher dose (1 mg/kg p.o.) in the SNI model using the same experimental paradigm described above. After administration of this dose 14 days after SNI, the extent of SNI-induced mechanical hypersensitivity was slightly, but not significantly, ameliorated as compared to vehicle-treated mice (Fig. 3c). By contrast, pregabalin, which in this experiment was given at 30 mg/kg p.o. [27], significantly inhibited the mechanical hypersensitivity in comparison to vehicle (Fig. 3c).

Finally, we assessed whether intravenous (i.v.) delivery of capadenoson affects SNI-induced mechanical hypersensitivity. For these experiments we used the same cohort of mice, but injected drugs 21 days after SNI (i.e., after a wash-out period of 7 days following the 1 mg/kg capadenoson p.o. measurements). We administered capadenoson at a dose of 0.3 mg/kg i.v. that significantly decreased infarct size in a model of acute myocardial infarction in rats [19]. As a control we used the full A_1_R agonist Cl-ENBA at a dose of 0.5 mg/kg that has been reported to inhibit SNI-induced mechanical allodynia in mice after i.p. delivery [28]. As shown in Fig. 3d, 0.3 mg/kg i.v. capadenoson did not affect SNI-induced mechanical hypersensitivity as compared to vehicle-treated animals. Unexpectedly, 0.5 mg/kg i.v. Cl-ENBA also failed to alter the mechanical hypersensitivity (Fig. 3d; see also discussion). Moreover, all mice treated with Cl-ENBA displayed obvious sedative effects within the first 30–45 min after drug injection. Altogether, these behavioral experiments suggest that capadenoson is of limited value for the treatment of neuropathic pain.

### Capadenoson fails to affect potassium currents in dissociated DRG neurons

**Fig. 4:**
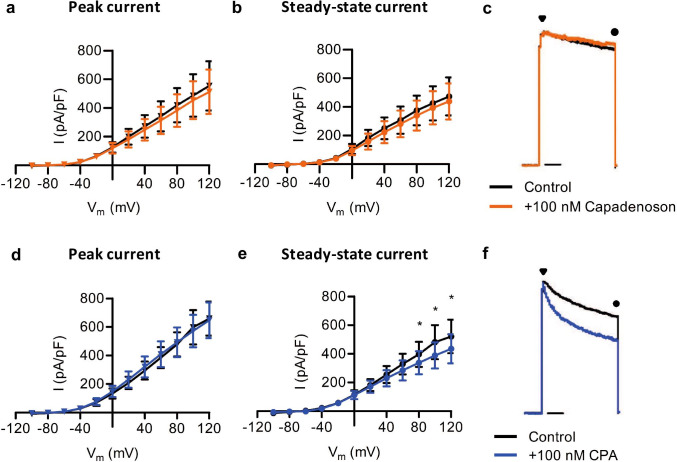
Potassium currents in DRG neurons are not affected by capadenoson. IV relations of the total potassium currents (I_K_) were measured in isolated lumbar DRG neurons of mice using the whole cell patch-clamp technique. After the measurement of the control current, the partial A_1_R agonist capadenoson (100 nM; n = 4; **a**–**c**) or the full A_1_R agonist N^6^-cyclopentyladenosine (CPA; 100 nM; n = 5; **d**–**f**) were added into the bath solution. Ten minutes thereafter, currents were measured again. Original registrations at + 100 mV are presented in **c** and **f** and indicate the peak current component (marked by a triangle) and the steady-state current component (marked by a circle)**.** Capadenoson failed to affect both the peak and steady-state currents. CPA did not affect the peak current but significantly reduced the steady-state current. Data are presented as mean ± SEM, *p < 0.05

We next assessed whether capadenoson affects electrophysiological properties of DRG neurons. Because coupling of A_1_R to neuronal potassium channels has been considered as a mechanism contributing to the analgesic activity of A_1_R agonists [29–31], we analyzed outward potassium currents (I_K_) of dissociated DRG neurons in presence of capadenoson. In particular, we assessed the transient, peak current component and the sustained, steady-state current component of I_K_ [32, 33]. Whole-cell patch-clamp recordings were performed at a holding potential of − 70 mV by applying a 1000-ms-long prepulse of -100 mV followed by series of 500-ms-long pulses ranging from − 100 to + 120 mV in intervals of 20 mV. As shown in Fig. 4, addition of capadenoson (100 nM) to the external solution did neither affect I_K_ peak currents (Fig. 4a) nor I_K_ steady-state currents (Fig. 4b). An original registration at + 100 mV is depicted in Fig. 4c. The I_K_ peak currents were also not affected by the full A_1_R agonist CPA (100 nM [13]; Fig. 4d). However, I_K_ steady-state currents were significantly reduced by CPA (Fig. 4e and 4f), pointing to a coupling of A_1_R and potassium channels in DRG neurons. The lack of effect of capadenoson in these experiments further supports the finding that pharmacological intervention with this partial A_1_R agonist might not be a valuable approach for the treatment of pain.

## Discussion

In this study, we investigated whether the partial A_1_R agonist capadenoson might be sufficient for treatment of neuropathic pain. Even though A_1_R is localized to DRG neurons and dorsal horn neurons, which are relevant for pain processing, capadenoson did not affect the pain behavior induced by peripheral nerve injury and chemotherapy, and it did not alter potassium currents in DRG neurons. Hence, capadenoson seems not to be suitable for neuropathic pain therapy.

The distribution of A_1_R in the nociceptive system has been investigated in earlier studies, however with conflicting results. In immunostaining experiments it was reported that A_1_R immunoreactivity is present in a subset of rat DRG neurons [10], and double-labeling experiments suggested that about 32%, 80% and 1% of A_1_R-positive rat DRG neurons co-express substance P, IB4 and NF200, respectively [11]. We here performed in situ hybridization experiments and confirmed A_1_R expression in a subset of mouse DRG neurons. However, our in situ hybridization combined with immunostaining revealed that A_1_R mRNA is expressed in 26%, 36% and 46% of neurons positive for CGRP, IB4 and NF200, respectively. Species differences in the distribution of A_1_R in DRG neurons of rats and mice, differences between protein and mRNA expression, or a lack of antibody specificity might account for this apparent discrepancy.

In the spinal cord, we found A_1_R mRNA to be enriched in dorsal horn neurons. This observation is in accordance with previous autoradiography experiments on rat spinal cord sections, in which A_1_R were detected predominantly in the superficial dorsal horn [34–36]. Immunohistochemical analyses of rat spinal cord sections were less consistent, because some studies showed a dense band of staining predominantly in lamina II of the dorsal horn [10, 12], whereas more diffuse A_1_R immunoreactivity throughout the spinal cord were observed in another study [13]. Hence, our in situ hybridization data add confidence that the major localization of A_1_R mRNA in the spinal cord are interneurons in the dorsal horn. It should be noted however that A_1_R has been detected in activated microglia cells after induction of neuropathic pain [37], and that delivery of an A_1_R agonist ameliorated the injury-induced microglia activation and neuronal sensitization [28]. Moreover, A_1_R are expressed in various supraspinal CNS regions including brain cortex, hippocampus, and cerebellum [9], which was however not investigated in this study.

Early preclinical studies in the 1980s with systemic and intrathecal administration of adenosine, A_1_R agonists and A_1_R antagonists suggested that targeting A_1_R might be suitable for treatment of neuropathic pain (for review, see [7, 38, 39]). However, the development of analgesics is hampered by the wide distribution of A_1_R leading to a variety of possible side effects, the high number of receptor subtypes (the four existing adenosine receptors present a sequence homology of 80–95% [9]), and the lack of truly subtype-selective agonists to be dispensed through clinically relevant routes of administration [31]. For example, systemic (intraperitoneal or intravenous) administration of the full A_1_R agonist CPA has been reported to inhibit neuropathic pain after nerve injury in rats [40, 41]. However, systemic treatment with CPA is accompanied by an intense depression of blood pressure as an unwanted side effect that might also affect the behavior in animal models of pain [40]. Moreover, A_1_R are thought to mediate the local anti-nociceptive effects of acupuncture, and injection of the A_1_R agonist 2-Chloro-CPA (CCPA) into the Zusanli point ST36 inhibited neuropathic pain in a model of peripheral nerve injury [42]. However, CCPA seems not to be suitable for systemic treatment as it reduced the latency to fall off the rotarod and caused catalepsy-like behavior in a dose-dependent manner [43]. Hence, the usefulness of systemic A_1_R agonists is limited by unwanted cardiovascular, motor and sedative side effects.

In general, improved efficacy of A_1_R agonists for pain treatment might be reached with allosteric modulators or partial agonists [7, 31, 44, 45]. Among the allosteric A_1_R modulators that have been tested for analgesia in vivo are T62 and TRR469. Oral administration of T62 reduced mechanical allodynia after peripheral nerve injury in rats. However, within 5 days of repeated daily administration, a tolerance occurred that led to decreased analgesic efficacy over time partly as a result of receptor down-regulation [46, 47]. T62 was also subjected to a clinical trial in patients with postherpetic neuralgia, which however was discontinued [9, 45]. Intraperitoneal administration of TRR469, a more potent allosteric A_1_R modulator as compared to T62, inhibited neuropathic pain in the model of streptozotocin-induced diabetic neuropathy and did not display locomotor or cataleptic side effects in mice [43]. Hence, there are obvious discrepancies on the effectiveness and tolerability of A_1_R agonists reported in different studies, probably due to the use of different compounds, routes of administration and models of neuropathic pain.

Another strategy to separate the desired from undesired pharmacological effects is to use partial A_1_R agonists. These compounds have been shown to activate only certain responses of A_1_R-mediated G-protein signaling, mostly in cells with high receptor reserve [48]. Moreover, the partial agonism is postulated to achieve conformational selection of a distinct active state, as recently shown for A_2A_R [49]. Hence, partial agonists might show better receptor selectivity due to increased receptor density and/or efficiency of receptor coupling to effector systems in the presence of nerve injury [7, 50, 51]. We here tested the partial A_1_R agonist capadenoson in two animal models of neuropathic pain. Capadenoson, which previously entered into two phase IIa clinical trials in patients with atrial fibrillation and stable angina, has been reported to have beneficial cardiovascular effects [19, 52, 53]. Unexpectedly, in our behavior experiments, four different doses of p.o. administered capadenoson (0.03, 0.1, 0.3 and 1 mg/kg) and one dose of i.v. delivered capadenoson (0.3 mg/kg) failed to significantly inhibit mechanical hypersensitivity in neuropathic pain models. In a previous study, p.o. administration of capadenoson at 0.3 and 1 mg/kg in mice led to a significant decrease of locomotion activity in a running wheel model as a surrogate marker for sedative CNS effects, and capadenoson at 1 mg/kg substantially decreased the running distance in this model by 37% [18]. We observed a trend towards inhibition of neuropathic pain behavior (not significant) at a dose of 1 mg/kg p.o. capadenoson in the SNI model. However, the profound impairment of running wheel behavior [18] indicates that this dose is associated with sedative adverse effects. Thus, we did not test higher doses of p.o. administered capadenoson in our study. Instead thereof, we assessed i.v. delivery of capadenoson at 0.3 mg/kg, but again did not observe any inhibition of neuropathic pain behavior. By contrast, capadenoson at 0.1 and 0.3 mg/kg i.v. has been reported to decrease infarct size in a model of acute myocardial infarction in rats [19]. Considering that capadenoson in the dose range of 0.3–1 mg/kg p.o. and 0.1–0.3 mg/kg i.v. provided significant pharmacological activity in other models [18, 19] we conclude that partial A_1_R agonism is of minor value for treatment of neuropathic pain. It should be noted however that in a recent study, capadenoson was found to have also activity at A_2B_R [54]. As A_2B_R agonists exhibit proinflammatory effects on immune cells [7], this might also affect the pain behavior after nerve injury or chemotherapy.

About the reasons for the ineffectiveness of the full A_1_R agonist Cl-ENBA in the SNI model we can only speculate. We intended to use 0.5 mg/kg Cl-ENBA as a positive control in our experiment with i.v. administered capadenoson, because Luongo and colleagues [28] reported that this dose significantly reduced neuropathic pain behavior after SNI. It has to be considered that Luongo et al. used a modified version of the SNI model, in which the tibial nerve is left intact [55], and not the sural nerve as in our study. Moreover, in the study by Luongo et al. Cl-ENBA was i.p. administered 7 days post injury and mechanical hypersensitivity was assessed with a dynamic plantar aesthesiometer using a ramp of 3 g/s [28], whereas we delivered Cl-ENBA i.v. 21 days post injury and used a ramp of 0.5 g/s. Although speculative, these differences in experimental settings might account for the discrepancies between the study of Luongo et al. and our findings.

The lack of efficacy of capadenoson for analgesia is also supported by our patch-clamp experiments, in which capadenoson failed to affect potassium currents in dissociated mouse DRG neurons. Various downstream mechanisms of A_1_R have been previously identified in different tissues including cAMP/PKA and PLC/IP_3_/DAG signaling, potassium channel stimulation, calcium channel inhibition, and β-arrestin mediated receptor modulation (for review, see [7]). In rat ventricular myocytes, A_1_R are coupled to potassium channels by G protein signaling [56], and we thus speculated that a coupling of A_1_R and potassium channels might also exist in DRG neurons. Indeed, in our patch-clamp experiments addition of the full A_1_R agonist CPA significantly decreased the steady-state potassium currents at positive voltage ranges. By contrast, the peak potassium currents were not altered, suggesting a specific inhibition of the sustained current component. In previous studies, adenosine and the full A_1_R agonist CCPA inhibited potassium currents by a steady-state block in AZF cells [33], and inhibition of sustained potassium currents by tetraethylammonium prolonged the duration of the repolarization phase and thereby reduced intrinsic firing in trigeminal neurons [32]. Accordingly, CPA has been shown to inhibit action potentials [57] and A- and C-fiber evoked field potentials [41] in neuronal tissues. Hence, we hypothesize that a decrease of the steady-state potassium current by CPA might lead to a reduced firing frequency in DRG neurons and inhibition of pain processing. However, in contrast to CPA, capadenoson did not affect the steady-state potassium currents in DRG neurons.

Altogether, in our study, a partial A_1_R agonist failed to ameliorate neuropathic pain in mice. If this holds true in other species or humans has to be shown.

## Data Availability

The datasets generated during and/or analyzed during the current study are available from the corresponding author on reasonable request.
